# In vivo study of corneal responses to increased intraocular pressure loading

**DOI:** 10.1186/s40662-015-0029-z

**Published:** 2015-12-10

**Authors:** Ahmed Elsheikh, Charles W. McMonnies, Charles Whitford, Gavin C. Boneham

**Affiliations:** School of Engineering, University of Liverpool, Brownlow Hill, Liverpool, L69 3GH UK; National Institute for Health Research (NIHR) Biomedical Research Centre, Moorfields Eye Hospital NHS Foundation Trust and UCL Institute of Ophthalmology, London, UK; School of Optometry and Vision Science, University of New South Wales, Sydney, Australia

**Keywords:** Corneal biomechanics, Topography, Numerical simulation

## Abstract

**Background:**

The cornea is responsible for two-thirds of the eye's refractive power which is a function of the shape and refractive index. The aim of this present study is to examine human eyes in vivo for corneal shape changes in response to short-term elevation in intraocular pressure.

**Methods:**

Videokeratographic and tonometric assessments at baseline were compared with the same assessments when intraocular pressure was elevated to approximately double (199 ± 22 %) the baseline levels using ophthalmodynamometer applanation of the sclera. Composite maps of the cornea and limbus were created by combining topographical assessments for central, nasal, temporal, inferior and superior fixation. Numerical finite-element simulations were custom built for each subject and the stiffness distribution across corneal surface modified to achieve matches between simulated and experimental data.

**Results:**

The stiffness distributions required to achieve simulation-experimental matches showed a consistent trend with the 2.5 mm annulus bounded by the limbus showing a mean stiffness reduction of 47.3 ± 10.8 % compared with the central cornea (*P* = 0.001).

**Conclusions:**

Corneal structure appears to provide the central cornea with a greater stiffness compared with the peripheral cornea and associated greater tolerance to elevation in intraocular pressure, consistent with the need for stable corneal refraction and vision. The method adopted to examine corneal biomechanical performance in vivo may have applications in additional studies.

## Background

Corneal biomechanical behavior is primarily a function of the stroma, which represents the bulk of corneal thickness, and is composed of lamellae of collagen fibrils embedded within a matrix of proteoglycans [1]. There are approximately 300 lamellae through the center of the normal cornea, running uninterrupted from limbus to limbus, rather like thin belts up to 0.2 mm broad and about 2 μm thick [2]. The number of lamellae increases by branching as the cornea thickens toward the periphery reaching about 500 at the limbus [3].

X-ray scattering has unambiguously demonstrated that the majority of collagen fibrils in the central region of human corneas adopt a preferred orientation in the inferior-superior (vertical) or nasal-temporal (horizontal) directions [4, 5]. The transition from meridional orientation at the center to circumferential at the limbus has been the subject of several studies, and most data indicate that the central meridional fibrils undergo a smooth transition to become circumferential as the limbus is approached [5–8]. At the limbus, these fibrils merge with the limbal annulus and possibly another population of circumferential fibrils originating from the sclera [5]. This change in collagen orientation from the apex to the limbus could be expected to lead to regional variations in biomechanical performance – in particular a reduction in corneal stiffness (increase in compliance) due to the reducing content of meridional fibrils in the transition zone [9] – and may affect corneal shape variation under changes in mechanical loading such as intraocular pressure (IOP) elevation.

The shape of the cornea is a function of a number of structural and biomechanical parameters, the most important of which are the material thickness and stiffness (as measured by the tangent modulus), as well as the IOP [10]. Examination of the biomechanical performance of the cornea with respect to shape has frequently been limited to ex vivo animal and human studies and by associated methodological deficiencies. These limitations have recently been reviewed [11].

The purpose of this study is to examine human eyes in vivo, explore corneal shape changes in response to short-term rises in IOP, and relate any such changes to possible regional variations in corneal stiffness. The study builds on earlier work, in which the central corneal shape was examined in normal human eyes under conditions of IOP elevated to a range of 374–443 % greater than normal [12]. Under this level of IOP elevation, the central corneal shape and refraction showed only minor changes. However, such studies using normal videokeratographic data collection methods can be limited to findings that do not allow analysis of significant areas of the peripheral cornea and may fail to detect regional differences in response. This study aims to extend videokeratographic data collection further into the peripheral cornea and to utilize the extended data, alongside numerical simulations of full eye globes, to determine the biomechanical performance of the transition peripheral zone compared with that at the center.

## Methods

Seven Caucasian participants with an age range of over 50 years were selected to include only healthy eyes with no history or evidence of corneal abnormalities or relevant systemic diseases.

### Experimental data

The methods used to increase IOP and monitor corneal topography in this study have been described and evaluated previously [13]. Baseline and elevated IOP were measured (mean of three readings for the left eye of each subject) using an Expert NCT Plus noncontact tonometer (Reichert Ophthalmic Instruments, Depew, NY), which was calibrated according to the manufacturer’s guidelines. A Medmont E300W computerized videokeratographic corneal topographer (Medmont Vermont, Victoria, Australia) was used to record baseline topography data for the same eyes. The topography assessment was based on elevation relative to a vertical plane that was tangential to the cornea at its front-most point and was determined independently of eye position. The instrument has a 32-ring placido cup target with the potential to provide corneal coverage from 0.25 mm to 11 mm diameter. For this instrument, manual alignment and automatically initiated image capture are combined with progressive accumulation and storage of the best three images. Each image is analyzed for centering, focus and stability, and then rated to yield a percentage figure for quality, after correction for detected defocus and alignment errors. Image ratings of over 75 % are deemed by the manufacturer to be acceptable [14]. However, image capture was continued in this study until three optimized images with a quality rating of at least 98 % were captured and retained for analysis. The remaining images were discarded. This procedure was intended to maximize measurement reliability.

After normal alignment readings were recorded, the measurements were immediately repeated with subjects instructed to alter fixation approximately 10° to the left and right and then down and up so that data from the peripheral cornea and the limbus could be recorded nasally, temporally, superiorly and inferiorly. The three sets of optimized corneal elevation data produced by Medmont software for each position were analyzed to derive the corresponding average topography, and the five average maps (one aligned and four with controlled misalignment) were processed mathematically as explained below to obtain a corneal map covering the whole corneal surface and the limbus-with map coverage diameter extending to 14 mm.

An ophthalmodynamometer (ODM) compressive force against the sclera was used to elevate IOP by application of the instrument foot plate to the adnexal skin. The 10 mm diameter metal foot plate was positioned on the temporal side under the lower lid, in line with the inferior palpebral furrow. The compressive ODM force applanates the sclera and the intraocular fluid displaced by the applanation is accommodated by increased distending forces on the sclera and cornea, and IOP is raised accordingly [15]. The IOP elevation varies with normal IOP and in proportion to the volume of the intraocular fluid displaced. The force delivered by the hand-held ODM approached a standard level by maintaining the same instrument setting, and a horizontal position for the force indicator throughout all measurements. Efforts to maintain the repeatability of the level of force delivered included the instrument footplate being applied tangentially to the scleral surface. By this means, an approximately equal distribution of the compressive force over the footplate area, and corresponding area of scleral applanation and associated force application were maintained for all measurements. Tonometry readings were repeated under this ODM force to assess the elevation in IOP induced during the videokeratographic assessments. All topographic assessments were then repeated with IOP elevated under the same ODM force and the same procedures described above for baseline measurements.

The study protocol was approved by the Human Research Ethics Advisory panel of the University of New South Wales, in accordance with the tenets of the Declaration of Helsinki. Ethics approval (number HC12467) was granted in response to this application. The purpose of the study was explained to all subjects who read and signed an informed consent form.

### Analysis of elevation data

The elevation data took the form of Cartesian point coordinates (x, y, z) with the center point of each data set located at (0,0,0). Central and peripheral maps of each eye were combined to cover the whole surface of the cornea and hence enable construction of an eye-specific numerical model. The map combination process relied on the introduction to peripheral maps of 3D displacements and rotations, which were optimized using the least squares method to reduce the difference in z coordinates between points on the peripheral maps and corresponding points on the central map with the same x and y coordinates. In order to overcome the discretized nature of the data and to enable the unrestrained matching of points on central and peripheral maps, central maps were fitted to Zernike polynomials such that the difference between the z coordinates between the two data sets could be obtained at any point (x, y) on the peripheral maps.

**Fig. 1:**
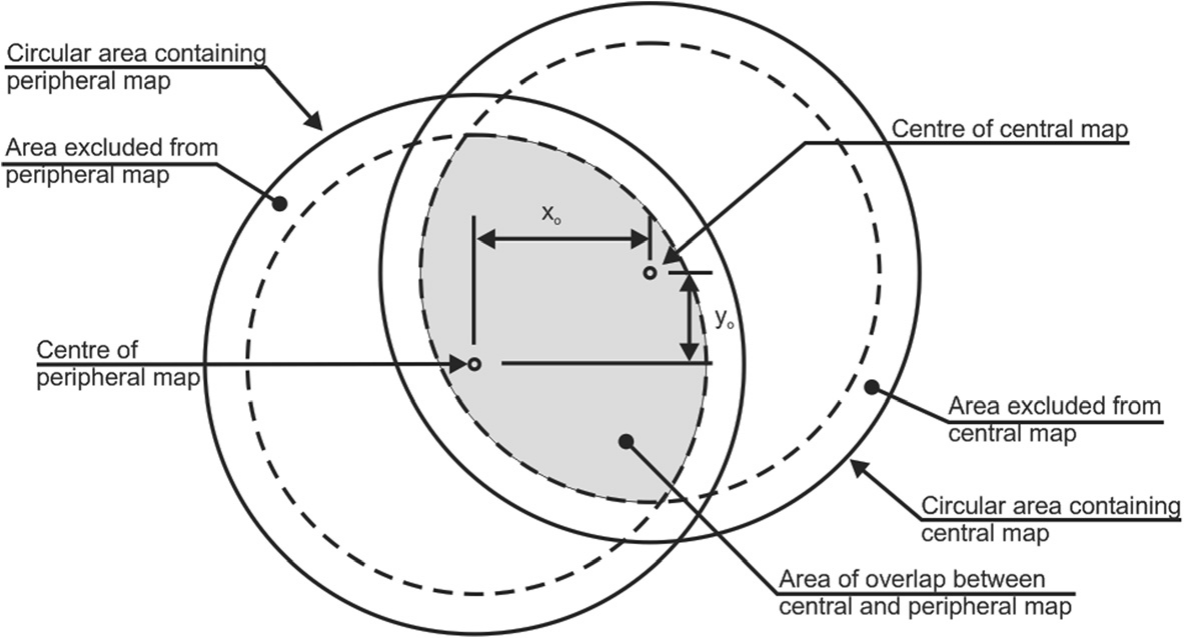
Combining central and peripheral maps – x_o_ and y_o_ are assumed displacement shifts in x and y directions

**Fig. 2:**
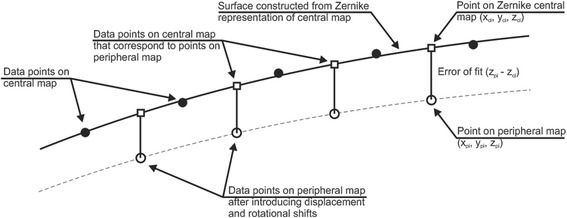
Fit between a central topography map and a peripheral map in a cross-sectional view

**Fig. 3:**
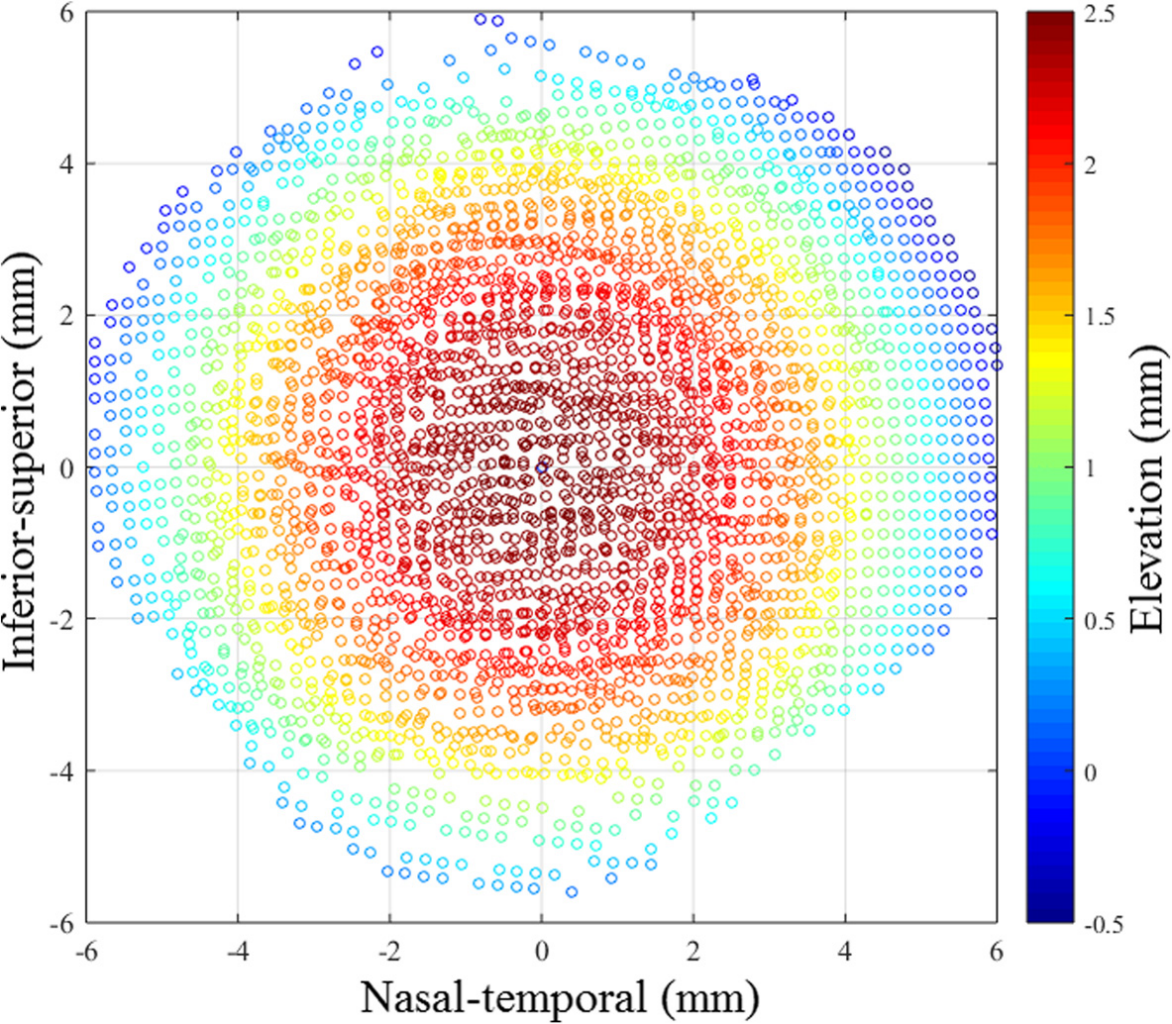
Example of measured corneal topography in its Cartesian form after the peripheral and central maps has been matched

Zernike polynomials of orders between 3 and 8 were attempted for all 7 central maps, and it was found that the error of fit reduced gradually with higher orders down to 1.5 ± 1.2 μm with eighth order Zernike polynomials, see Table 1. Initially, all data points were included in this step. However, due to the relatively larger fitting errors at the outer-most points [16, 17], it was decided to exclude the points within an outer ring with 0.8 mm width (approximately 16 % of all points), reducing the error of fit from 1.5 ± 1.2 to 0.5 ± 0.4 μm. Each of the corresponding peripheral data sets were then matched to the central set by first excluding the edge data similar to the central maps, then making an initial assumption of a reasonable shift involving 3 displacements (x_o_, y_o_, z_o_) and 3 rotations (α, β, γ), Fig. 1. The area of overlap between the peripheral and central sets was determined according to the X and Y coordinates. Points in the peripheral set within the area of overlap were identified, and for each point i (x_pi_, y_pi_, z_pi_) a corresponding point on the central map (x_ci_ = x_pi_, y_ci_ = y_pi_, z_ci_) was located using the Zernike polynomials, Fig. 2. Figure 3 provides the topography in the form of Cartesian points after the matching has been performed. The error of fit (z_pi_ − z_ci_) was calculated for all points within the overlap area and the root mean square (RMS) of error $\sqrt{\frac{1}{\mathrm{n}}{\displaystyle \sum_{\mathrm{i}=1}^{\mathrm{n}}}{\left({\mathrm{z}}_{\mathrm{pi}}-{\mathrm{z}}_{\mathrm{ci}}\right)}^2}$, where *n* = number of points on the peripheral map within the overlap area with the central map, was determined. The least squares method was then employed to reduce the RMS of error of fit to a minimum while changing the values of the 3 displacements and 3 rotations introduced into the peripheral map. The values of the RMS of error had an average and standard deviation values of 4.5 ± 2.4 μm. After fitting all four peripheral maps, one by one, to their corresponding central map, all five data sets were again fitted to eighth order Zernike polynomials, and in this step the average error of fit was 2.6 ± 1.4 μm.

**Table 1:** Error of fit with Zernike polynomials with orders between 3 and 8 for central maps and combining central and peripheral maps together

	Zernike RMS of error (μm)	Map combination RMS of error (μm)		
Zernike order	Whole central map	Reduced area of central map^a^	Whole maps	Reduced area maps*
3	9.5 ± 5.6	4.2 ± 2.2	14.1 ± 9.9	9.0 ± 6.3
4	2.9 ± 3.2	0.8 ± 1.4	11.6 ± 8.9	7.6 ± 6.7
5	2.4 ± 2.4	0.6 ± 1.5	11.5 ± 9.2	7.2 ± 7.1
6	1.9 ± 1.6	0.6 ± 1.2	11.6 ± 7.8	6.6 ± 6.0
7	1.8 ± 1.6	0.8 ± 0.7	10.6 ± 7.2	5.1 ± 3.6
8	1.5 ± 1.2	0.5 ± 0.4	10.1 ± 5.6	4.5 ± 2.4

^a^ Area of maps excluding data contained with an edge area with 0.8 mm width

In order to assess the accuracy of this procedure, z coordinates were calculated for points within a central circular area with 10 mm diameter using the two sets of Zernike polynomials derived respectively for the central map alone and for the five maps combined. The average differences between the two sets of z coordinates remained below 2.4 μm in all seven corneas.

### Numerical study

Numerical finite-element simulations were custom-built for each of the seven eyes included in this study. The simulations included the whole ocular outer tunic (cornea and sclera), and aimed to provide a close representation of in vivo conditions of each eye including the precise adoption of the anterior topography under baseline conditions as well as the value of the normal IOP. However, assumptions had to be made to enable consideration of the non-uniform thicknesses of the cornea and sclera and the regional variation of material properties within the sclera. The corneal and scleral thicknesses were assumed to vary linearly from 545 μm at the corneal apex to 695 μm at the limbus, 590 μm at the equator and 960 μm at the posterior pole [18–21]. The material properties of the sclera were varied gradually from highest stiffness at the limbus to lowest stiffness at the posterior pole, based on the results of an earlier experimental study and as discussed below [21].

**Fig. 4:**
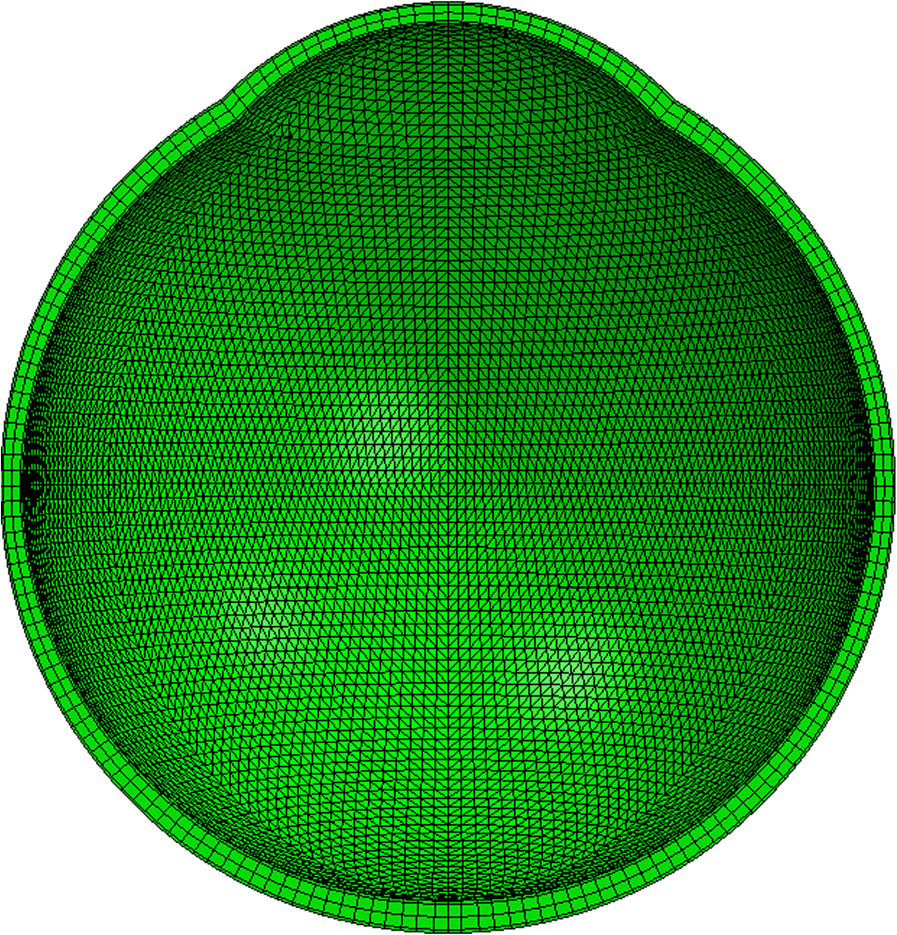
Cross-section of a numerical model of the eye globe. Model constructed with 100 element rings, 17 of which are in the cornea and 4 layers; providing 1, 2 and 1 layer/s for the epithelium, stroma and endothelium, respectively

Based on the findings of earlier studies, the simulations used 120,000 six-noded solid elements, arranged in 4 layers and 100 rings, 17 of which were in the cornea, and connected with 75,010 nodes [22], see Fig. 4. While full contact was assumed between the element layers representing the sclera, the weak corneal inter-lamellar adhesion necessitated reducing the adhesion level between the layers of elements representing the cornea to the level observed experimentally in a previous study [23]. The models were restrained against movement in the Z direction at all equatorial nodes on the external surface and in the X and Y directions at all nodes along the models’ longitudinal axis.

The simulations were designed such that each corneal ring could have different material constants relating to a common constitutive material model, while the sclera was allowed to have only three sets of material constants defining the anterior, equatorial and posterior regions, which extended within rings 18–44, 45–72, and 73–100, respectively; as defined from a previous study [21]. All material models were hyperelastic of the form [24–26]: σ = a(e^bε^ − 1), where σ and ε were the stress and strain, and a and b were constants. Differentiating this equation with respect to strain provides a linear relationship between the tangent modulus, E (a measure of material stiffness) and the stress: $\mathsf{E} = \mathsf{d}\mathsf{\sigma}\kern0em /\kern0em \mathsf{d}\mathsf{\varepsilon } = \mathsf{a}\ \mathsf{b}\ {\mathsf{e}}^{\mathsf{b}\mathsf{\varepsilon }} = \mathsf{b}\left(\mathsf{\sigma} + \mathsf{a}\right)$, which shows that b is the slope of the E-σ relationship. Changes in the stiffness between regions could therefore be introduced through proportional changes in the value of b for the different simulations examined.

The material model used in all corneal stromal elements was controlled first according to the relevant subject’s age (in years) based on the results of an earlier experimental study [26], which produced values for parameters a and b according to the expressions:

$$ \begin{array}{l}\mathsf{a}=\mathsf{35}\times \mathsf{1}{\mathsf{0}}^{\hbox{-} \mathsf{9}}\mathsf{a}\mathsf{g}{\mathsf{e}}^{\mathsf{2}}+\mathsf{1.4}\times \mathsf{1}{\mathsf{0}}^{\hbox{-} \mathsf{6}}\mathsf{a}\mathsf{g}\mathsf{e}+\mathsf{1.03}\times \mathsf{1}{\mathsf{0}}^{\hbox{-} \mathsf{3}}\mathsf{a}\mathsf{nd}\kern1em \\ {}\mathsf{b}=\mathsf{0.0013}\mathsf{a}\mathsf{g}{\mathsf{e}}^{\mathsf{2}}+\mathsf{0.013}\mathsf{a}\mathsf{g}\mathsf{e}+\mathsf{99}.\kern1em \end{array} $$

The models for the scleral regions were derived by taking b_anterior_ = 3 × b_central_cornea_, b_equator_ = 0.88 × b_anterior_ and b_posterior_ = 0.85 × b_anterior_, based on earlier experimental findings [21, 27, 28]. The stress-strain relationships for different parts of the eye model (including individual cornea rings) were created in a Visual Basic tool based on the values of the a and b parameters. While parameter a depended only on the participant’s age and hence remained unchanged, b was allowed to vary widely and the resulting stress-strain relationships for different parts of the eye were introduced in the analysis files used on the Abaqus software program. The tool was designed to conduct inverse modelling procedures wherein the material properties in different parts of the eye were initially set assuming all cornea element rings had the same stress-strain behavior and that sclera properties followed the trends explained above. The tool used the Particle Swarm Optimization (PSO) method [29] to change the stress-strain relationship of each model segment in the cornea (by changing b parameters) while adopting the objective function: $\sqrt{\frac{1}{n}{\displaystyle {\sum}_{i=1}^n}{\left({z}_{numerical,i} - {z}_{experimental,i}\right)}^2} = \mathsf{min}$, where *n* = number of anterior nodes on cornea model. The PSO method started with widely-spaced 69 points (particles), each given a “position” or a set of 17 values of b between 0.1 and 10 the initial value of b_central_cornea_ for the 17 cornea element rings. The fitness (value of objective function) of each particle was calculated and used to move the position (or change the b values) to a new position that was expected to have better fitness using the following equation: $\mathsf{B}\left(\mathsf{t} + \mathsf{1}\right) = \mathsf{B}\left(\mathsf{t}\right) + \mathsf{v}\left(\mathsf{t} + \mathsf{1}\right)$, where B(t) and B(t + 1) are the b values of the particles at the current and new iteration, and v(t + 1) is a velocity term derived as:

$$ \mathsf{v}\left(\mathsf{t}+\mathsf{1}\right)=\left(\mathsf{w}\times \mathsf{v}\left(\mathsf{t}\right)\right)+\left({\mathsf{c}}_{\mathsf{1}}\times {\mathsf{r}}_{\mathsf{1}}\times \left(\mathsf{P}\left(\mathsf{t}\right)\hbox{-} \mathsf{B}\left(\mathsf{t}\right)\right)\right)+\left({\mathsf{c}}_{\mathsf{2}}\times {\mathsf{r}}_{\mathsf{2}}\times \left(\mathsf{G}\left(\mathsf{t}\right)\hbox{-} \mathsf{B}\left(\mathsf{t}\right)\right)\right). $$

In this equation, P(t) vector represents the particle’s best position (with best fitness) found so far, G(t) vector is the best known position found by any particle in the analysis so far, and w, c_1_, c_2_, r_1_, r_2_ are constants taken as 0.7, 1.4, 1.4, 0.5, 0.6, based on Microsoft’s MDSN guidance. The analysis was repeated for up to 10,000 iterations while monitoring the fitness of the 69 particles considered, and this was sufficient for the analysis to converge on a best solution (min fitness error) in all cases.

## Results

The mean age for the 7 subjects (4 males) was 39.4 ± 17.2 years (range 17–68 years), Table 2. The normality of this sample is indicated by means, standard deviations and ranges for the central curvature, astigmatism and differences between inferior and superior curvature, which were 42.94 ± 0.61 D (42.29–43.90 D), 0.77 ± 0.44 D (0.60–1.82 D) and 0.14 ± 0.54 D (−0.50 to 0.79 D), respectively. The average central corneal radius of curvature and the shape factor (measurement of deviation from a sphere where a shape factor of 1 equates to a perfect sphere) were 7.957 ± 0.156 mm and 0.665 ± 0.037 mm before IOP elevation, and 7.948 ± 0.149 mm and 0.695 ± 0.034 mm after IOP elevation. The standardized ODM force induced a 98.7 % mean elevation of IOP (Table 2), which is similar to the 99.4 % finding obtained in another study of normal subjects [13], which used the same ODM compressive force setting.

**Table 2:** Individual increases in IOP for the 7 Subjects

Subject	Gender	Age (years)	Baseline IOP (mmHg)	Post ODM IOP (mmHg)	% Increase
1	F	21	12.2	27.4	124.6
2	M	17	13.1	22.3	70.5
3	M	47	14.7	31.2	112.2
4	M	68	24.4	42.6	74.6
5	M	43	17.1	32.7	91.2
6	F	50	13.3	29.5	121.8
7	F	30	20.7	40.5	95.7
Average		39.4	16.5	32.3	98.7
SD		17.9	4.5	7.1	21.7

*IOP* = Intraocular pressure, *ODM* = ophthalmodynamometer

**Fig. 5:**
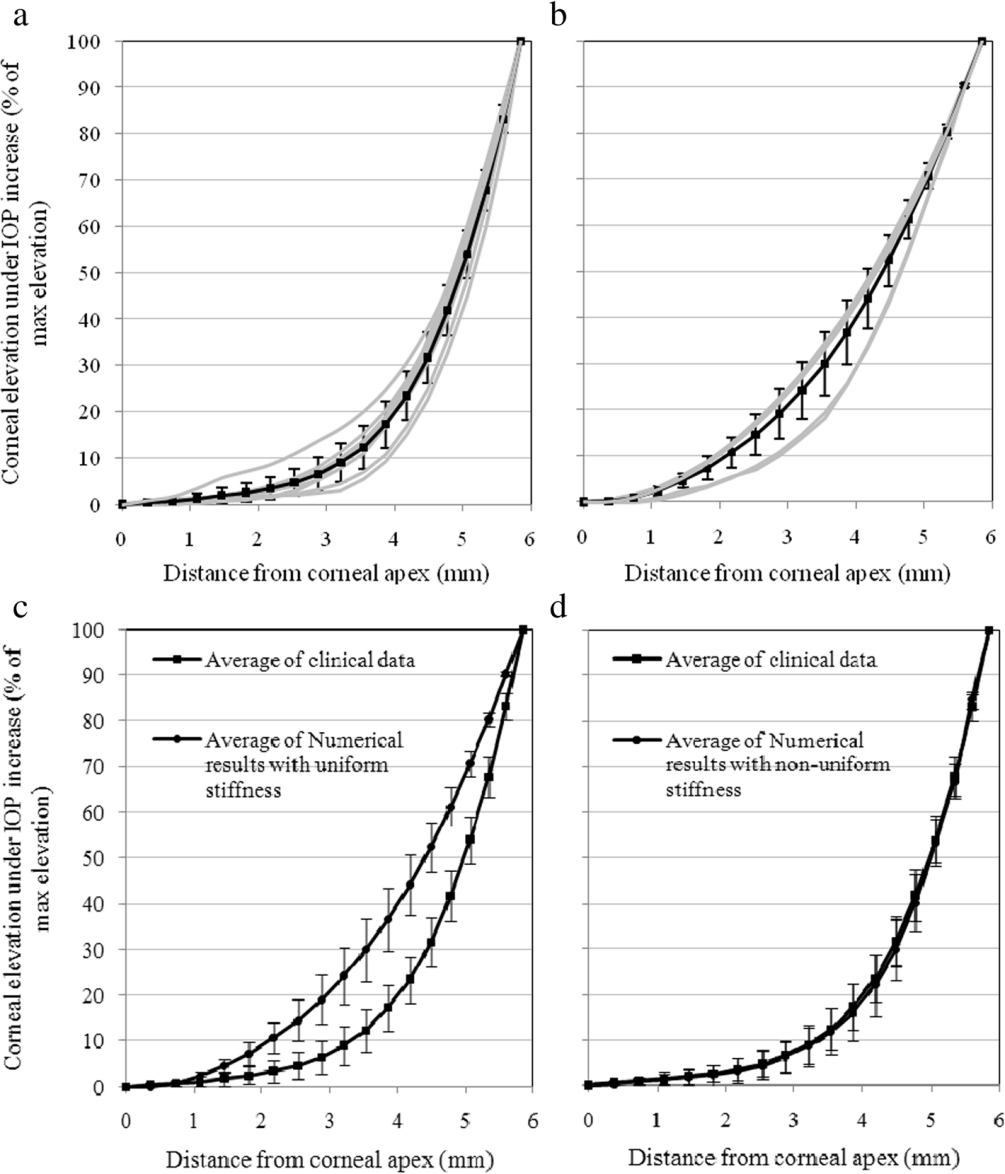
Elevation data presented as percentage values of maximum elevation. **a** Experimental data for all seven corneas. **b** Numerical data assuming uniform corneal stiffness for all seven corneas. **c** Comparison between average experimental and uniform-stiffness numerical data. **d** Comparison between average experimental and non-uniform stiffness numerical data with error bars depicting standard deviation values – light lines show individual results while heavy lines depict average distributions

The simulation study started with all corneal elements given the same material model. The resulting distributions of corneal deformation under the increase in IOP were compared to the corresponding experimental data and found to follow a considerably different trend. Figure 5 shows the distribution of corneal deformation for all 7 participants as obtained experimentally and predicted numerically, while assuming uniform corneal stiffness. In all cases, the experimental data showed a clear concentration of deformation in the 2.5 mm wide corneal circumferential annulus next to the limbus, compared with more uniform distribution in the numerical results, as can be seen in Fig. 5c. The average differences in corneal elevations under IOP increase are also given for individual eyes in Table 3 and shown to be large; up to 11 μm or 13 % of the maximum corneal elevation.

**Table 3:** Differences in corneal elevation between the experimental and numerical data as obtained from uniform-stiffness and non-uniform-stiffness simulations

Participant	Simulations based on uniform stiffness distribution	Simulations based on non-uniform stiffness distribution
	(μm)	(μm)
1	11.1 ± 7.4 (0.1–20.9, 13.0 %)	0.5 ± 0.4 (0.0–1.4, 0.5 %)
2	10.2 ± 7.0 (0.2–20.7, 11.0 %)	3.4 ± 3.3 (0.1–8.7, 3.6 %)
3	6.3 ± 4.7 (0.5–12.7, 8.1 %)	1.4 ± 1.0 (0.2–2.8, 1.8 %)
4	2.4 ± 2.2 (0.1–6.3, 4.2 %)	0.8 ± 0.5 (0.0–1.4, 1.4 %)
5	9.8 ± 6.4 (0.2–17.6, 12.2 %)	1.5 ± 1.5 (0.1–4.6, 1.8 %)
6	6.0 ± 4.6 (0.1–12.4, 9.0 %)	0.3 ± 0.4 (0.0–1.5, 0.5 %)
7	6.9 ± 4.5 (0.1–12.3, 13.6 %)	0.8 ± 0.7 (0.1–2.3, 1.6 %)

Results include the average, standard deviation and range in microns, and the average as a percentage of the maximum experimental corneal elevation for each eye

**Fig. 6:**
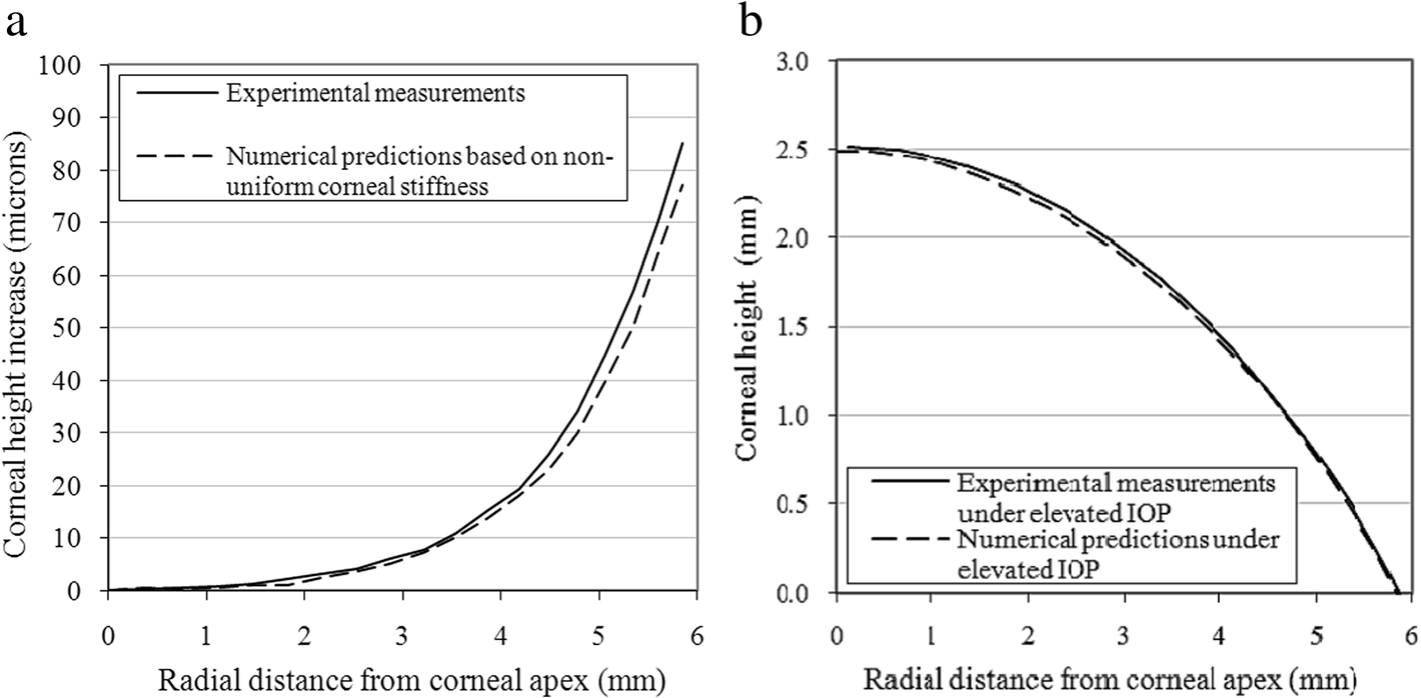
Example of a match between the experimental elevation data (following IOP increase). The numerical predictions are presented after adjustment of stiffness distributions for Participant 1. **a** Comparisons of corneal deformation. **b** Comparisons of corneal topography along the temporal-nasal center-line

Based on these results, the numerical simulations were modified such that individual corneal rings were assigned different material models until the numerical deformation distributions closely matched the experimental data. The least squares method was used to assess the quality of the match while reducing the sum of the squared errors of fit, *Σ* (*Z*_*experimental*_ – *Z*_*numerical*_)^2^, to a minimum. The close match with the experimental data is demonstrated in Fig. 5d and Table 3 where the average differences in corneal elevation caused by IOP increase are shown to be up to 3.4 μm or 3.6 % of maximum corneal elevation, which is considerably lower than for the analyses with uniform stiffness distribution. An example of the close match obtained with non-uniform stiffness distribution for subject 1 is shown in Fig. 6.

**Fig. 7:**
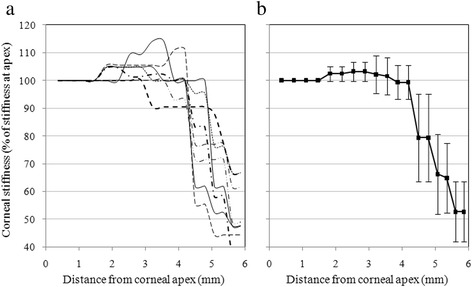
Corneal stiffness distributions as obtained numerically for all 7 participants. **a** individual participants. **b** average distribution for all participants with error bars representing the values of standard deviations

The material stiffness distributions that had been required to achieve close match in all 7 eyes examined are depicted in Fig. 7, and show a consistent trend in which the stiffness in the 2.5 mm wide circumferential zone bounded by the limbus has a considerably lower value compared with the apex. The average behavior presented in Fig. 7b shows a stiffness reduction in this zone of 47.3 % compared with the apical stiffness, with a standard deviation (SD) of 10.8 % (*P* = 0.001). There is also a small increase in stiffness of about 3.3 ± 6.7 % in the paracentral zone formed by an annulus lying between rings with radii of between 2.0 and 3.5 mm (*P* = 0.016).

## Discussion

Corneal biomechanical properties are heterogeneous, highly anisotropic, non-linear and viscoelastic [30]. Accordingly, the tangent modulus of the cornea varies directionally and regionally, such that a high modulus is exhibited meridionally in the central and paracentral areas, and circumferentially at the limbus, due to the specific arrangement of collagen fibrils [31]. The change of fibril orientation from meridional to circumferential, which takes place within an annulus approximately defined by ring diameters 8.0 and 11.5 mm, suggests a possible material stiffness reduction in this transition zone. This reduction was thought to be partially compensated by a peripheral thickness that is greater than central thickness [32], which may be due to the stromal lamellae interweaving, braiding and branching to form new lamellae on different layers in the peripheral cornea [3, 33]. However, interweaving and braiding could affect the tissue’s tensile stiffness, which is more relevant to the biomechanical action of the cornea under the effect of IOP. In engineering applications, braiding has been found to reduce the tensile stiffness of composite materials by more than 30 % [34]. Similarly, surgically created braiding of connective tissue led to reductions in tangent modulus (a measure of stiffness) of tendons used in the treatment of cruciate ligament injury [35]. Although the effect of braiding has not been studied in corneal tissue, it is possible that it leads to similar reductions in stiffness in the corneal periphery. The expected lower tissue stiffness and the resulting concentration of IOP-induced deformation at the transition zone are compatible with the observation of little noticeable change in refractive power with changes in IOP such as found previously [12]. This type of response appears to be a natural method to ensure stable refractive power is maintained in normal eyes despite significant changes in IOP, which may occur over limited time periods [36].

The current study attempted to quantify the reduction in material stiffness of the corneal periphery that might be caused by both the reduced content of meridional fibrils arrangement of stromal lamellae. The study used topography data obtained in vivo for the left eyes of 7 participants subjected to a temporary IOP elevation. Topography maps, covering the entire cornea and limbus, were obtained before and after the increase in IOP and analyzed to determine the profile of resulting corneal deformation. Nonlinear finite-element simulations of the seven eyes were constructed and used to estimate the stiffness distribution across the corneal surface that would be required to lead to deformation profiles similar to those obtained experimentally. Although the study involved a small number of participants, the findings were quite similar across the sample and indicated a significant reduction in material stiffness in a circumferential zone close to the limbus with a width of approximately 2.5 mm. The stiffness reduction in this region was consistent; down by an average of 47.3 ± 10.8 % (*P* = 0.001). These results are comparable with the findings of an earlier study on excised human corneas placed under posterior distending pressure and found to have reduced meridional stiffness in the 1 mm wide ring of tissue close to the limbus [37]. Compatible results were also obtained in in vitro studies on human and bovine corneas, which concluded that the tissue’s tangent modulus was higher at the central and paracentral regions and lower at the periphery [11, 38].

The evident correlation between the stiffness distribution across corneal surface and the corresponding stromal microstructure points at the need to consider either the stiffness variation as found in this study or the collagen fibril density and orientation in the finite-element simulations of corneal behavior. Assumptions of corneal homogeneity may lead to unacceptable approximations in behavior prediction.

The methodology used in the study has some potential limitations. The experimental procedure used may have resulted in some deformation of eye shape but uniformity of responses throughout the 360° of the corneal periphery suggests that this effect was not significantly apparent in the cornea and may have only been restricted to the applanated area of the sclera. The assumed corneal thickness may have an effect on the outcome. However, any error in the assumed central corneal thickness is likely to be reduced when the relative central corneal thickness and peripheral corneal thickness are considered. As it is the relative stiffness that was considered, this assumption is not considered to have a significant effect on the conclusions of the study. Maintaining sclera properties with three discrete regions could affect the results of the study. These effects are limited due to the relatively high stiffness with respect to the cornea.

## Conclusion

The study is an example of how in vivo measurements could yield useful information on corneal biomechanical behavior and provide an alternative to ex vivo tests, which while being increasingly reliable still have the disadvantage of unquantified differences in behavior between ex vivo and in vivo tissue.

The topography changes associated with short-term intraocular pressure elevation in human eyes were used in finite-element numerical simulations and showed that corneal periphery had lower stiffness than the central region.
